# The Community Research Liaison Model: Facilitating community-engaged research

**DOI:** 10.1017/cts.2023.31

**Published:** 2023-03-10

**Authors:** Christina Jäderholm, Jessica Currier, Kim Brown, Ariane Audett, Laura Campbell, Steven Blakesley, Lynda Crocker Daniel, Sylvia Miller, Sara Mishalanie, Chelsea Ruder, Jackilen Shannon

**Affiliations:** 1 Oregon Clinical and Translational Research Institute, Oregon Health & Science University, Portland, OR, USA; 2 Knight Cancer Institute, Oregon Health & Science University, Portland, OR, USA; 3 Oregon State University, USA

**Keywords:** Community engagement, rural health, community liaison, community research, community capacity

## Abstract

The Community Research Liaison Model (CRLM) is a novel model to facilitate community-engaged research (CEnR) and community–academic research partnerships focused on health priorities identified by the community. This model, informed by the Principles of Community Engagement, builds trust among rural communities and expands capacity for community and investigator-initiated research. We describe the CRLM development process and how it is operationalized today. We followed a multi-phase process to design and implement a community engagement model that could be replicated. The resulting CRLM moves community–academic research collaborations from objectives to outputs using a conceptual framework that specifies our guiding principles, objectives, and actions to facilitate the objectives (i.e., capacity, motivations, and partners), and outputs. The CRLM has been fully implemented across Oregon. Six Community Research Liaisons collectively support 18 predominantly rural Oregon counties. Since 2017, the liaison team has engaged with communities on nearly 300 community projects. The CRLM has been successful in facilitating CEnR and community–academic research partnerships. The model has always existed on a dynamic foundation and continues to be responsive to the lessons learned by the community and researchers. The model is expanding across Oregon as an equitable approach to addressing health disparities across the state.

## Background

Academic–community relationships can support the design and conduct of research responsive to local needs and priorities and build lasting change through education and increased research capacity. In this article, we will describe how we developed and implemented a Community Research Liaison Model (CLRM), a community-engaged research (CEnR) approach to respond to rural needs in Oregon and build sustained capacity for CEnR and data-driven solutions to local health issues. The CLRM is a joint effort led by our National Clinical and Translational Science-funded Clinical and Translational Research Institute and National Cancer Institute-funded comprehensive cancer institute.

CEnR (and the closely related Community-Based Participatory Research; CBPR) emerged in the 1940s and has evolved over time across fields such as psychology, sociology, public health, and social work [1]. Central to CEnR is the role of communities as partners and active participants in all aspects of knowledge creation through research activity [2]. The social justice movement was instrumental in informing the principles around CEnR, viewing lived experiences, historical harm, and social structures as important factors for community wellness and health promotion [1]. CEnR has become increasingly used for translational and implementation sciences aiming to take research “from bench to practice,” a shift is in part due to national funding priorities [3]. Multiple frameworks guiding community-engaged implementation and adaptation of evidence-based interventions have been suggested since 1967, with over 50 added since the turn of the millinum [4]. Finally, CEnR has been suggested for supporting advocacy and equitable health policy work by providing a frame for centering multiple and diverse voices [5]. When health research aims to make a sustainable impact, practice and policy change may often be an end goal.

Part of ensuring better health is including community values, processes, and lived experience as contextual factors for understanding health risks and barriers to health promotion [6]. Long-term changes have far more viable prospects when the community defines the problem, contributes knowledge, receives training, and participates in and co-structures research activities [7,8].

In this article, we describe the structures and processes that guide our work applying CEnR in Oregon, and how it may be replicated across other regions. We also provide examples specific to local communities in Oregon as a reflection of the research and health promotion resulting from our process.

## Model Development

The inception of our model was guided by the principles of community engagement and an acknowledgement that all communities have existing community-based organizations involved in health promotion efforts [9]. The institutional support from a large academic entity facilitated the development and continues to be the backbone for the day-to-day operations of what has become a formal model for CEnR in Oregon.

To develop our model, we followed a multi-phase process with continuous iterations and lessons-learned sessions to design and implement an effective community engagement model that could be replicated. Our work began with a pilot in the three largely rural counties of Central Oregon. We hired a community engagement specialist, a member of the local community, to examine community research engagement feasibility and readiness. The specialist implemented an adapted version of the Interactive Systems Framework (ISF) [10], a research-to-practice framework embracing community engagement. We chose the ISF because there are several notable examples in the literature where this framework was used to guide research to practice to community engagement activities [11–13]. Importantly, the ISF recognizes that a top-down approach to community engagement implementation is suboptimal [4,10]. Key stakeholders and community partnerships are integral to an effective, long-lasting approach to community engagement. Our adaptation of the ISF began by developing the Community Research Engagement Assessment (CREA) to explore community motivation.

The ISF is based on the equation: *R=MC2* (*R*eadiness = *M*otivation × General *C*apacity and Intervention-Specific *C*apacity), where readiness refers to the extent to which the region is willing and able to implement, participate in, and direct CEnR and use data to drive decision-making [14]. The CREA assessed community motivation (i.e., local champions and leadership), general capacity (i.e., community infrastructure and resources), and intervention capacity (i.e., adoption of research-based practices and data-driven decision-making). We collected data through key informant interviews, gleaning information about the region, the general capacity of organizations that focus on improving health and conducting research, and the capacity within the community/organization for using data to inform decisions and conduct research. We assessed motivation for building research capacity, academic partnerships, and the idea of a research advisory. We also gathered community perceptions of our academic institution to understand the level of recognition and trust the institution carried in the community. We interviewed leaders across several sectors including government, education, business, health and human services, community advocacy, and bioscience/biotechnology as part of the assessment.

Through the assessment, we identified gaps, current efforts, and opportunities where community-based organizations could be supported by resources, including academic expertise, research services, funding opportunities, technical assistance, and training. Further, the community engagement specialist identified champions and leaders in the community to be part of an advisory group to assure efforts are aligned with community needs and priorities.

**Fig. 1.**
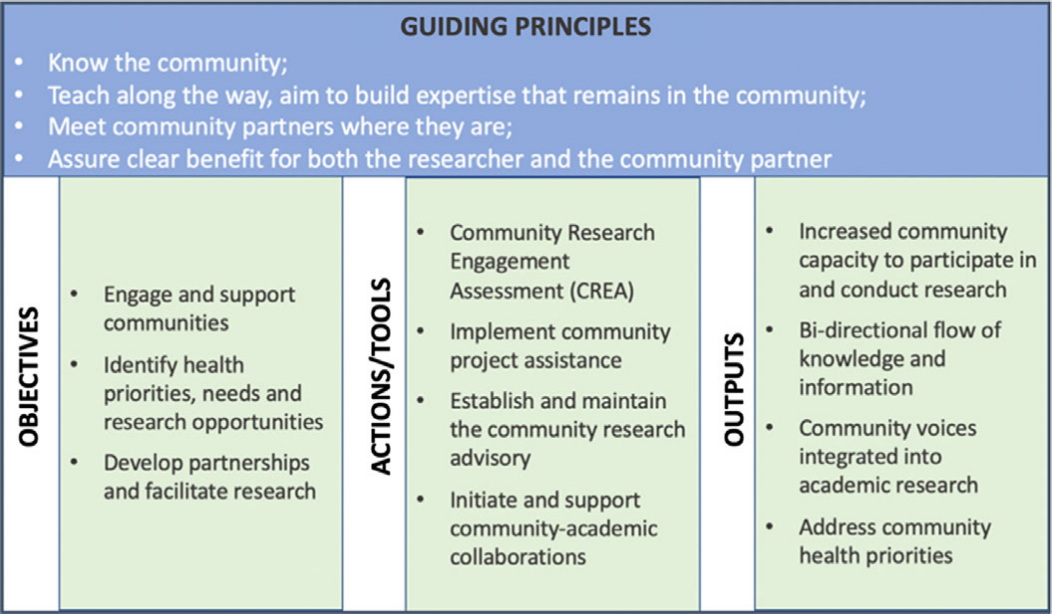
Community-engaged research conceptual framework.

Using results from the CREA, and informed by the ISF and initial efforts of the community engagement specialist, we created a new conceptual framework to guide our formal model. Our conceptual framework specifies the guiding principles, objectives, actions to facilitate the objectives (i.e., capacity, motivations, and partners), and outputs (Fig. 1). Our conceptual framework fits within the ISF but lays out the specific steps and tools we identified as useful to understand local needs and moving to action. The CREA continues to be an important part of actions used to engage with communities, but other actions and assessment tools have been added.

Building on the success of the initial community engagement specialist in Central Oregon, we moved toward expanding this model to other rural regions in our state and the engagement specialist role was renamed “Community Research Liaison” (CRL). The model which we use today is therefore known as the CRLM.

### The Community Research Liaisons

The CRLs are the lynchpin of our CRLM. CRLs are embedded in their community and work with community partners following a consistent yet flexible approach to identify and respond to the health-related needs of their region. The CRLs are full-time employees hired from their community. They have a bachelor degree or higher and a background in public health, community organizing or nonprofit work, as well as strong interpersonal and communication skills. Liaisons often have only minimal prior experience with academia or research, but have a deep connection to their local community. They develop and maintain relationships with local partners, allowing for a bidirectional exchange of ideas and support. The CRLs will be assessing trust in their community, but do not yet have pre-post assessments.

CRLs promote collaborations between community and academic researchers and facilitate research-based practices and data-driven decision-making. The result is increased capacity in a community’s ability to participate in, and conduct, research activities.

The CRLM builds upon the science or implementation of CEnR through a novel long-term relationship building approach. The CRL is embedded in the community in which they live and work. The literature suggests that effective CEnR is built over time [15,16]. This approach facilitates relationship building and trust through proximal exchanges where trust is established through successive collaborations and partnership opportunities.

### Community Research Hub

To provide support and assist in maintaining fidelity to the CRLM, our CRL team is supported by a coordinating center, the Community Research Hub (The Hub). The Hub is a centralized office strategically located in the center of the state and is funded in part by its university affiliation, and in part by projects. The Hub employs a dedicated community research program manager that leads the liaison team and supports the liaisons by connecting regional partners to academic and other resources. The Hub provides resources, technical assistance, and guidance in support of the liaison activities to facilitate CEnR. Further, The Hub supports the community research program manager to identify areas of possible common interests across CRL sites and to leverage resources to efficiently address needs that have been identified across multiple regions.

### The Community Research Liaison Model

The CRLM has four actions or “tools” which CRLs and academic researchers use to move from objectives to outputs within communities (Fig. 1). We outline these actions below, describing how they support community involvement in research activities. Action 1 (CREA) determines the community’s readiness to engage in research. Depending on the findings from the assessment, the remaining three actions may be carried out, or only a subset of actions.

### CRL Model Action 1. Community Research Engagement Assessment

An important first step in establishing the role of a CRL within the community is the completion of a CREA. As described above in the description of the Central Oregon pilot, the CREA is a community-based tool to understand specific community capacity and readiness to engage in research. The assessment can be led by CRLs and community members. The CREA is based on interviews with key partners or leaders in the community identified based on their role in health care, education, the public sector, and other industry or business unique to the particular region. The interviews follow the same format as the Central Oregon pilot. The findings are used to guide development and priorities of a community advisory group, to identify needs that may be addressed through new partnerships, and to learn about beliefs, knowledge, and resources within the community that may inform the CRL’s work.

### CRL Model Action 2. Community Project Assistance

To facilitate consistent community engagement and support, all CRLs have implemented a common approach for community-based organizations to receive research technical assistance, training, and explore funding opportunities. This approach, the community project assistance program, is where community–academic partnerships often develop and where needs are identified. CRLs conduct standardized consults with community-based organizations and groups to better understand their research and data needs. Consultation notes are reviewed weekly with The Hub leadership team to identify opportunities for connecting the organization with the appropriate resources, technical assistance, and academic collaborators. After a connection is made, CRLs continue to serve as facilitators, bridging the gap between community and academia, and building the capacity of community organizations to participate in and conduct CEnR.

### CRL Model Action 3. Support Community Research Advisory

CRLs also coordinate and facilitate a community research advisory. The purpose of developing a research advisory is to bring local and regional community leaders together with academic partners to collaborate, network and build local capacity for research and data-driven decision-making to improve health and wellness in the community. The advisories most often identify, develop, and champion larger cross-sector projects. Each advisory has a slightly different structure to meet the unique needs and priorities of the community and is comprised of community leaders who represent the diversity and interests in each region. The model of engagement is based on collaboration and shared leadership and ensures the community is an active part of the research process and provides input on priorities for engagement.

### CRL Model Action 4. Develop Community–Academic Collaborations

Through the community project assistance and community research advisories, the CRLs facilitate community and academic connections. The CRLs understand and are able to communicate their community’s health priorities to academic researchers and conversely, the researcher’s objectives and strategy to the community. CRLs foster these relationships by facilitating consistent bidirectional communication, creating opportunities for discussion, framing relevant questions and concepts, identifying funding opportunities, and drafting scopes of work or protocols.

## Results

The CRLM is now fully implemented in one urban and four rural areas in Oregon that include the North Coast (3 counties), the South Coast (2 counties), Southern Oregon (2 counties), and the Columbia River Gorge (4 counties) and continues in the three counties of Central Oregon. A CRL has also recently been hired to work with the four counties of Northeast Oregon.

**Fig. 2.**
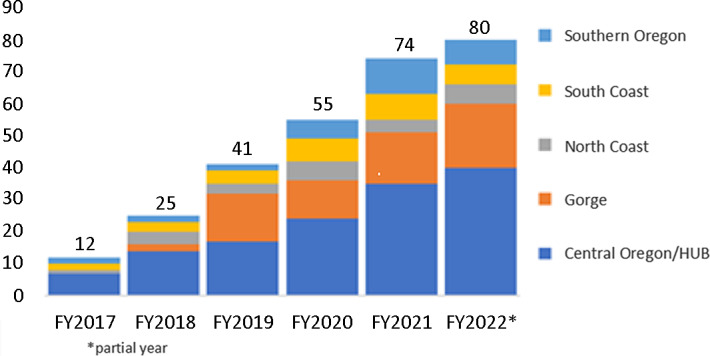
Number of projects by year and region.

The success of the initial Central Oregon pilot is seen in Fig. 2. The CRL began supporting projects in 2017, and the number of projects has grown each year. As the CRL model was initiated in each additional region, projects in those areas have also increased. Overall, since 2017, the liaison team has engaged with communities on nearly 300 community projects.

Projects have a strong focus on rural areas (44 projects), reach diverse populations (6 with tribes, 5 with Hispanic or Latino organizations), and different age ranges (26 with youth/children, 7 with older adults). CRLs facilitate CEnR supported by a range of team members, including data analysts, student workers and interns, project managers, and researchers from the Community Research Hub. Multiple services are provided for a project and can range from assistance with literature searches, to quantitative and qualitative data collection, data analysis, interpretation and presentation, program evaluation, and survey design interpretation and presentation.

At the inception of the CRLM, most projects were pro-bono in an effort to build trust and demonstrate the value of partnership. Over time, more projects have been funded by contracts and grants, coming both from the community and researchers.

### Case Studies

The selected case studies (Perinatal Care Coordination example and Table 1) provide context for the types of projects and relationships facilitated by the CRLM. The CRLM facilitates community engagement and support through the actions and tools outlined in our conceptual framework (i.e., CREA, Community Project Assistance, Community Research Advisory, and Community–Academic Collaborations) and follow a consistent approach to community engagement through these actions and tools. The impact of this work is particularly notable when CRLs identify areas of shared need across regions and are able to connect partners across the state or use work in one region to inform projects elsewhere.

The selected case studies vary in focus and scope, but all address local priorities and health needs and follow the CRLM starting with Action 1 followed by various combinations of Actions 2, 3, and 4.

### Perinatal Care Coordination, a Case Study

Central Oregon’s Perinatal Care Coordination (PCC) is a community-developed and public health-led cross-sector partnership to assist pregnant and postpartum individuals in Central Oregon’s three rural counties to access health insurance, perinatal care, nutritional services, behavioral health services, and other referrals with ease and dignity. PCC’s objectives include 1) decrease incidence of low birth weight in Central Oregon; 2) increase timely perinatal care initiation; 3) increase eligible referrals to community-based resources, WIC, and behavioral health services; and 4) solicit provider and community partner feedback on the implementation process and sustainability of the program.

*Objectives.* Through prior completion of the CREA (Action 1), the CRL was aware of a group that met regularly to address perinatal care and began attending, listening, and getting to know the group participants and their work. While attending, the CRL also worked to accomplish a second objective of identifying health priorities, needs, and research opportunities. The CRL identified a need when the PCC program shared that to sustain funding and expand program reach, it was necessary to evaluate the program and demonstrate intervention efficacy. The PCC team needed technical assistance to develop and implement an evaluation.

*Actions.* The CRL shared information about the Community Project Assistance Program (Action 2) with the community group, who expressed interest in collaborating. The CRL conducted a consult to obtain necessary information and to determine how The Hub could best support the project. A doctoral student and her mentors, who had interest and expertise in maternal and child health, were identified as potential academic collaborators. The CRL initiated a collaboration between the researchers and the PCC team by making introductions, scheduling meetings, and facilitating conversations to plan program evaluation and means to transition the model into other sites (Action 4).

*Outputs.* Through this process, CRLs developed a new partnership, and the community partners increased their capacity to participate in and conduct research. The work began by discussing the necessary components to create evidence for an intervention and conduct a research project. The academic–community team worked together to design a research protocol and began to identify potential funding. A Ph.D. student led the work with help from academic mentors and an undergraduate intern. They identified an evaluation framework, submitted three grants, and secured $30,000 in partial funding to begin the project. They have successfully completed planned evaluation activities and designed a handbook to aid transition of the intervention into other regions.

### Table 1 Description

The case studies in Table 1 are organized by the CRL actions and tools: community project assistance, community research advisories, and community–academic collaborations. While distinct, CRL actions are interrelated. Outputs from one action could inform another action, or two actions might run in parallel and supplement each other. For example, health needs identified through a CREA (Action 1) lead to areas addressed through the community project assistance program (Action 2), and/or informs priorities for community research advisories (Action 3). Within each Action category, Table 1 presents projects by: Partners, Objectives, Actions, and Outputs.

**Table 1.** Facilitating and building capacity for community-engaged research case studies

Community Project Assistance			
*Partners*	*Objective*	*Actions*	*Outputs*
Organization – South Coast Equity Coalition Location – Southern Oregon coast Population – Rural residents	Build a coalition to enhance health equity in Coos and Curry counties through advocacy, amplifying voices of underrepresented communities, community education, and identifying gaps in services and access	• Brought community partners and organizations together to develop a coalition • Provided diversity, equity and inclusion (DEI) education and trainings to community members • Identified strengths and gaps in achieving regional health equity	• Developed a marketing and outreach plan to follow when introducing the coalition to the community • Organized and hosted a local DEI conference • Partners secured a $5,000 grant to hire a consultant to facilitate the development of a mission, vision, and goals
Organization – Council on Aging Location – Central OregonPopulation – Older adults	Improve understanding of social isolation and loneliness among older adults in Central Oregon and develop and implement a plan to decrease these feelings among the target population	• Identified validated measurement tools and develop a survey to assess social isolation and loneliness • Designed or adapted, implemented, and evaluated an intervention to decrease feelings of social isolation and loneliness	• Designed a 40-item tool to measure social isolation and loneliness and administered 102 surveys • Designed, implemented, and evaluated an intervention to decrease loneliness and social isolation, a program that connects volunteers and older adults to create opportunities for socialization and needs identification • Submitted 5 grants and secured $138,000 in funding to implement and evaluate the program as a pilot and then as a sustained program • Enrolled 38 participants in the program, volunteers and program participants. • Completed 447 calls totaling 10,765 minutes, with an average of 24 minutes per call • Participants reported decreased feelings of social isolation and loneliness and general satisfaction with the program • Prior to the program 70% of participants indicated they felt *isolated* “some of the time” or “often” compared to 50% after completing the program • Prior to the program 79% of participants indicated they felt *left out* “some of the time” or “often” compared to 24% after completing the program • Developed a new academic–community partnership and engaged three master’s level students in the project
Organization – Klamath Tribal Health Location – Southern Oregon Population – Klamath Tribal members experiencing food insecurity or at risk of experiencing food insecurity	Increase access to fresh produce by providing fresh fruits and vegetables in a Community Supported Agriculture (CSA) box and nutrition education for Klamath Tribal members experiencing food insecurity or at risk of experiencing food insecurity	• Partnered with a local CSA to develop a produce prescription program for Klamath Tribal Health • Developed a system to deliver produce boxes to Tribal members along with nutrition and cooking education brochures • Designed and conduct cooking classes for participants	• Increased access to fresh vegetables and nutrition knowledge for 40 tribal families who are food insecure or at risk of being food insecure by providing 10 boxes of fresh produce with educational materials per family • Decreased food insecurity. After the program, participants felt less worried about food running out before having money to buy more (60% indicated they were worried before the program and 40% after) • 57% of participants noticed an increase in energy levels and physical activity duration • 43% of participants reported a decrease in blood sugar levels and blood pressure after completing the program • All participants agreed or strongly agreed that they were satisfied with the produce variety and overall program • Engaged two master’s level and one undergraduate student in the project
Organization – Bridging the Gap Location – Central Oregon Population – Individuals with behavioral health needs	Increase care coordination between primary care and specialty behavioral health providers to improve behavioral health care	– Developed data collection tool to facilitate interagency collaboration and communication to improve behavioral health care – Established an academic–community partnership to design a protocol to assess effectiveness of care coordination– Established long-term academic partnership between Bridging the Gap, a group representing primary care, behavioral health, and public health, and OHSU Biostatistics and Design Core	– Coordinated referrals for 337 patients from 3 primary care to 2 specialty behavioral health organizations– 80% of patients had an appointment offered within two weeks of the referral date– 55% of patients completed at least 4 visits within 60 days of referral– 28% of patients who completed at least 1 appointment had a note sent back to their primary care organization within 30 daysOf 160* patients who completed 4 visits with a behavioral health provider within 60 days of referral, 89% (142 people) were still engaged at the end of data collection period * This number excludes 44 people who had less than 60 days in the data collection period
Community Research Advisories			
*Partners*	*Objective*	*Actions*	*Outputs*
Group – Community Health Action and Advocacy and Resource Team (CHART) Location – North Oregon Coast Population – Clatsop County residents and public health partners	Work collaboratively with community members to impact policy, systems, and environmental changes, and improve health equity to raise the overall health and wellness of all county residents	Established a multi-sector community collaborative focused on improving the health and well-being of Clatsop County residents Applied a collective impact model to create alignment among programs and opportunities to collaborate on public health projects across agencies, organizations, and systems Acted as incubator for project development and implementation (i.e., The Nutrition Consortium, The Extraordinary Living Conference, Way to Wellsville, “I Will Walk 2,000 Miles”) Served as forum for public health policy initiatives (i.e., Tobacco Retail License Ordinance, Tobacco-Free Campus)	Convened monthly meetings for sustained community collaborations and networking opportunities for 10 years; Representatives from 100 community organizations regularly attend meetings to discuss opportunities for leveraging and collaborating on health and wellness work Established a rapid response COVID-19 Task Force to respond to emerging situations during the COVID-19 pandemic Hosted 3 annual free Place Matters Clatsop County conferences starting in 2019 to acknowledge and learn about the intersection of place and health and bring diverse voices to the table to celebrate strengths in the community and identify and act on opportunities to improve community health Recognized by Clatsop County Board of County Commissioners for the important role CHART has played in raising the level of health in Clatsop County and observed their 10^th^ anniversary with a formal proclamation
Group – Healthy Klamath Location – Southern Oregon Population – Klamath County residents and public health partners, rural	Guide community health improvement in six priority areas including food security, housing, maternal and child health, oral health, physical well-being, and suicide prevention	Established a multisector partnership that guides community health improvement efforts, formed in response to consistently low rankings in the annual Robert Wood Johnson Foundation (RWJF) County Health Rankings Established a community collaborative made up of representatives from community-based organizations and community members to improve health, create livable spaces, address community needs, and share ideas and resources Launched initiatives, programs, and policy changes to achieve coalition goal	A 50-member collaborative with dedicated staff Implemented workgroups addressing six priority areas: food security, housing, maternal & child health, oral health, physical well-being, and suicide prevention Completed projects on topics such as tree planting, implementing protected bike lanes, breastfeeding needs assessment Achieved certified Blue Zones Community® status One of four 2018 winners nationwide to be awarded the Culture of Health Prize
Organization – Healthy Klamath Trends on Tots Workgroup Location – Southern Oregon Population – Pregnant women	Reduce infant mortality and associated risk factors	• Established a system to track missed prenatal appointments for case management follow-up • Gathered, created, and translated layperson information on causes of infant mortality and smoking cessation	• Implemented Direct On-Scene Education (DOSE) training, an intervention to decrease sleep-related infant death using first responders • Safe Sleep Gold Standard Certification pursued by birthing center, resulting in policy and systems change and community safe sleep education • Decreased preventable infant deaths within the 1^st^ year of life from 10 per 1,000 live births in 2017 to 7.8 in 2020 • Distributed educational materials to the community
Community–Academic Collaborations			
*Partners*	*Objective*	*Actions*	*Outputs*
Organization – Central Oregon Research Coalition Location – Central Oregon Population – Central Oregon (Deschutes, Crook, and Jefferson counties) residents and public health partners	Strengthen data driven decision making and research-based practices to improve the health and well-being of Central Oregonians	• Established a community coalition, led by a steering committee, to connect Central Oregonians and provide opportunities to advance research-based practices and data-driven decision making • Established an academic–coalition partnership to support high impact, community-driven projects that advances the coalition’s objectives	• A coalition of 11 steering committee members representing academia, early childhood education, public health, and nonprofit organizations, and 242 general coalition members • Supported 3 public health projects on homelessness, older adult health, and data education • Hosted 2 networking events for community members (paused due to COVID-19 interruptions)
Organization – Columbia Gorge Postpartum Support Location – Columbia River Gorge Population – Postpartum families	Increase access to relief strategies to try to improve mental health for postpartum families through inclusive postpartum doula care and lactation consultations	Established an academic partnership between the organization and Oregon Health & Science University researchers Conducted a needs assessment Developed a strategic plan for the organization Developed local capacity to deliver postpartum doula care	Developed a research protocol to examine the effectiveness of doula care on reducing postpartum mood disorders Submitted 9 grants and secured 5 totaling $52,200 Hired 3 lactation consultants Delivered 5 weekly postpartum and breastfeeding support groups in 7 counties Delivered postpartum care to 82 clients totaling 149 appointments for services including initial lactation consultation, follow-up lactation consultation, prenatal consultation, postpartum doula visit, and telehealth lactation follow-up

## Discussion

Similar to other community programs dedicated to building capacity for CEnR [17], our CRLM is based on the idea that community partnerships build research capacity at the community level and are the backbone for pursuing equitable solutions and better health for the communities we serve. Our model is unique in its use of CRLs to facilitate community–academic partnerships; this model has brought both successes and challenges over the years. An important aspect of the model is that community partners build and sustain their capacity to lead and conduct research, even after projects conclude. Partners build skills such as data literacy and identification of evaluation frameworks; these carry across time and projects as communities identify new or expanded needs. Understanding local, unique community contexts, both rural and urban, is a defining feature of our CRLM and is what leads us to build and continue to strengthen research capacity and community relationships. Another aspect of research capacity is the ability to identify and formulate research questions to move specific health promotion efforts faster and more effectively in the desired direction (like pursuing funding, or establishing the evidence base for new programs). Often, one project leads to the next as community members and partners find use and value in data-driven approaches (see Council on Aging in Table 1). As the CRLM continues to grow in capacity and reach (geographically and demographically), the lessons learned along the way are used for consistent reflection and improvement.

### Lesson 1. The Value of Being Present

CRLs live within the communities that they serve, have deep roots in the social fabric, and understand customs, beliefs, and community processes. The consensus among CRLs is that trust and relationship building is a long-term endeavor and being embedded in their community is a fundamental feature of the model. To that end, CRLs have discovered the value of “participating beyond their own projects.” A key aspect of building strong relationships is attending meetings, public forums, or small group settings which are not required, expected, or necessarily relevant to current work. However, at times activities outside active projects have created tension between research agendas and timelines and the more elusive, long-term investment in building relationships. Liaisons sometimes struggle to quantify their work, but as stronger research advisories have been formed and communities have become more aware of the opportunities to collaborate with CRLs and The Hub, and therefore more active in requesting technical assistance, the value of their less quantifiable work has become more apparent.

### Lesson 2. Communicating Expectations

CRLs also participate in health promotion, intervention design, and program maintenance. This role has led to the facilitation of new projects or inquiries but has also helped shape how communities have engaged with results and findings. CRLs are often well positioned to frame research results in a practical way that resonates with communities or local organizations. However, in facilitating community dissemination or engaging in new inquiries, CRLs have also found themselves grappling with the somewhat limited scope of research in comparison with community expectations. Although community inquiries can have a narrow scope of health promotion, much of the local interest is often rooted in social justice and structural changes. The negotiation between the scope of research, intervention design, and the community’s long-term goals is rarely in opposition, but CRLs have had to develop skills and language tools to more clearly communicate expectations bidirectionally between community and researchers.

CRLs and the formation of regional advisories are an effective strategy to increase research capacity and respond to community-identified population health priorities. The model consists of four distinct actions: 1) Assess Community Research Engagement Readiness; 2) Implement the Project Assistance Program; 3) Support Community Research Advisories; and 4) Develop Community-Academic Collaborations. The model has been implemented in six rural regions in Oregon. Community research capacity was bolstered by program evaluations, data collection and analyses, dissemination, and implementation research. Programmatic activities ranged from maternal and child health to social inclusion for older adults. As a result of implementing the CRLM, our institution has dramatically increased the number of successful academic–community collaborations, enhanced community capacity to address health-related concerns, and established lasting trusted relationships.
